# Irgm proteins attenuate inflammatory disease in mouse models of genital *Chlamydia* infection

**DOI:** 10.1128/mbio.00303-24

**Published:** 2024-03-19

**Authors:** Jacob Dockterman, Jeffrey R. Reitano, Jeffrey I. Everitt, Graham D. Wallace, Meghan Hendrix, Gregory A. Taylor, Jörn Coers

**Affiliations:** 1 Department of Molecular Genetics and Microbiology, Duke University Medical Center, Durham, North Carolina, USA; 2 Department of Immunology, Duke University Medical Center, Durham, North Carolina, USA; 3 Department of Pathology, Duke University Medical Center, Durham, North Carolina, USA; 4 Geriatric Research, Education, and Clinical Center, VA Health Care Center, Durham, North Carolina, USA; 5 Department of Medicine, Division of Geriatrics, and Center for the Study of Aging and Human Development, Duke Universitygrid.26009.3d Medical Center, Durham, North Carolina, USA; National Institute of Allergy and Infectious Diseases, Bethesda, Maryland, USA

**Keywords:** *Chlamydia*, immunity-related GTPases, disease tolerance, interferons, immunopathology, IRGM, sexually transmitted diseases

## Abstract

*Chlamydiae* are obligate intracellular bacterial pathogens that may cause genital pathology via induction of destructive host immune responses. Human-adapted *Chlamydia trachomatis* causes inflammatory disease in human hosts but is easily cleared in mice, and mouse-adapted *Chlamydia muridarum* establishes a productive and pathogenic infection in murine hosts. While numerous anti-chlamydial host resistance factors have been discovered in mice and humans alike, little is known about host factors promoting host fitness independent of host resistance. Here, we show that interferon-inducible immunity-related GTPase M (Irgm) proteins function as such host factors ameliorating infection-associated sequalae in the murine female genital tract, thus characterizing Irgm proteins as mediators of disease tolerance. Specifically, we demonstrate that mice deficient for all three murine Irgm paralogs (pan-*Irgm*^−/−^) are defective for cell-autonomous immunity to *C. trachomatis*, which correlates with an early and transient increase in bacterial burden and sustained hyperinflammation *in vivo*. In contrast, upon infection of pan-*Irgm*^−/−^ mice with *C. muridarum*, bacterial burden is unaffected, yet genital inflammation and scarring pathology are nonetheless increased, demonstrating that Irgm proteins can promote host fitness without altering bacterial burden. Additionally, pan-*Irgm*^−/−^ mice display increased granulomatous inflammation in genital *Chlamydia* infection, implicating Irgm proteins in the regulation of granuloma formation and maintenance. These findings demonstrate that Irgm proteins regulate pathogenic immune responses to *Chlamydia* infection *in vivo*, establishing an effective infection model to examine the immunoregulatory functions and mechanisms of Irgm proteins.

**IMPORTANCE:**

In response to genital Chlamydia infection, the immune system mounts a proinflammatory response to resist the pathogen, yet inflammation must be tightly controlled to avoid collateral damage and scarring to host genital tissue. Variation in the human IRGM gene is associated with susceptibility to autoinflammatory diseases but its role in ameliorating inflammatory diseases caused by infections is poorly defined. Here, we use mice deficient for all three murine Irgm paralogs to demonstrate that Irgm proteins not only provide host resistance to Chlamydia infections but also limit associated inflammation in the female genital tract. In particular, we find that murine Irgm expression prevents granulomatous inflammation, which parallels inflammatory diseases associated with variants in human IRGM. Our findings therefore establish genital Chlamydia infection as a useful model to study the roles for Irgm proteins in both promoting protective immunity and limiting pathogenic inflammation.

## INTRODUCTION

*Chlamydia trachomatis* is an obligate intracellular bacterial pathogen that can cause a spectrum of human diseases. Chronic or repeated infection with *C. trachomatis* serovars D-K can cause genital scarring and pelvic inflammatory disease, infertility, and an increased risk of ectopic pregnancy (1). Ocular serovars A–C cause corneal inflammation and blinding trachoma (2), and serovars L1–L3 can cause lymphogranuloma venereum (3). All of these chlamydial disease syndromes are mediated largely by destructive host immune responses. Genital and ocular chlamydial diseases comprise a massive worldwide public health burden, and no protective vaccine exists (4).

Infectious disease manifestation is determined by protective resistance mechanisms that reduce infectious burden as well as tolerance mechanisms that ameliorate the pathogenic impact of an infection independent of burden relief (5). Animal models of genital *Chlamydia* infection have proven instrumental in elucidating both protective and pathogenic host immune responses (6). *Chlamydia trachomatis* is able to persist and cause disease in its preferred human host, but it is readily cleared in murine hosts and generally does not cause disease (7). However, *C. muridarum* has adapted to subvert murine mechanisms of cell-autonomous immunity and therefore establishes a productive infection in the mouse host (8–10). Genital infection with *C. muridarum* results in a pathogenic inflammatory response and genital scarring similar to that observed in humans with chlamydial disease (11–13). Pathology in genital *C. muridarum* infection is dependent on certain murine host immune factors such as CD8+ T cells, neutrophils, IL-1, and TNF-α (6). Therefore, while host immunity is necessary to clear *Chlamydia* infection and provide disease resistance, regulation of pathogenic immune responses is necessary to prevent genital scarring and thus confer tolerance to *Chlamydia* disease. Several correlative studies have associated a number of the same immune mediators with human disease in *C. trachomatis* infection, suggesting that murine *C. muridarum* infection is a useful model for human chlamydial disease (14–18).

As the pathology in genital *Chlamydia* infection is mediated largely by host immunity, *Chlamydia* infection provides a viable model to study mechanisms of immunoregulation. In humans, immunity-related GTPase clade M (IRGM) is a protein whose functions and mechanisms remain to be fully elucidated, which is linked with a variety of inflammatory diseases such as inflammatory bowel disease (19), sepsis (20), and autoimmune thyroid disease (21). Analysis of disease-related *IRGM* variants mostly correlate with decreased gene expression, implying that IRGM functions to limit inflammation (22). Mechanistically, human IRGM has been implicated in regulating autophagy and may regulate inflammasome activity via selective autophagy of inflammasome machinery (23–25). However, the mechanisms by which variants in *IRGM* lead to human inflammatory disease remain to be understood.

Mice possess three paralogs of IRGM (Irgm1, Irgm2, and Irgm3), which were initially thought to primarily control cell-autonomous immunity to intracellular pathogens such as *C. trachomatis* (26). Murine Irgm proteins regulate the localization and activity of a subset of “effector” GKS IRG proteins, which are expressed upon stimulation with the cytokine gamma-interferon (IFNγ) and traffic to intracellular pathogens to mediate their destruction via partially understood mechanisms (27–31). In the absence of Irgm1 and Irgm3, IFNγ stimulation leads to mislocalization and cytoplasmic aggregation of GKS IRGs and a failure to target and restrict the growth of most intracellular pathogens in an IFNγ-dependent manner (30, 32, 33). *Irgm1*^−/−^ mice are defective for immunity to virtually all intracellular bacterial and protozoal pathogens, while *Irgm3*^−/−^ mice are susceptible to a more limited set of pathogens (30, 33, 34). Interestingly, for a small number of examined intracellular pathogens such as *Listeria monocytogenes*, *Irgm1*^−/−^ mice are defective for IFNγ-mediated resistance while concurrent deletion of Irgm3 in *Irgm1*/*m3*^−/−^ mice reverses this susceptibility, implying complex regulatory relationships among Irgm proteins in some contexts (33, 35, 36). With respect to *C. trachomatis*, both *Irgm1*^−/−^ and *Irgm3*^−/−^ mice are defective for IFNγ-mediated resistance to bacterial infection. *Irgm1*/*m3*^−/−^ cells are also defective for host resistance *in vitro* and display increased bacterial burden *in vivo* early in genital infection, although an enhanced T cell response correlates with clearance of the infection over time (32, 37). The other remaining paralog, Irgm2, has not been studied in *Chlamydia* infection but only in the context of infection with the protozoan pathogen *Toxoplasma gondii*, where it acts as a critical host resistance factor (38, 39).

While murine Irgm proteins are critical for defense against most intracellular pathogens, humans lack Irgm-regulated effector IRGs and human IRGM has only been shown to contribute to host defense against a small subset of pathogens such as *Mycobacterium tuberculosis* and not against *Toxoplasma* or *Chlamydia* (40, 41). Mouse and human IRGM proteins therefore appear to only share limited functional homology in cell-autonomous immunity; however, murine Irgm proteins—especially Irgm1—have been shown to regulate inflammation in contexts similar to human IRGM. *Irgm1*^−/−^ mice display susceptibility to intestinal inflammation in multiple contexts (42–44), increased cytokine production upon LPS treatment (45, 46), and type I interferonopathy resembling Sjogren’s syndrome (47), paralleling several human disease syndromes associated with *IRGM* variants. Irgm1 also has been shown to regulate T cell homeostasis, with *Irgm1*^−/−^ mice displaying lymphoid collapse upon infection with certain bacterial pathogens including *C. trachomatis*, although additional deletion of Irgm3 appears to reverse this phenotype (37, 48, 49). On a cellular level, Irgm1 appears to regulate inflammasome activation via selective autophagy, similar to human IRGM (23), and defects in mitophagy can result in spontaneous interferonopathies and linked susceptibility to bacterial infections (47, 50). Irgm2 on the other hand has been shown to limit noncanonical inflammasome activation (51, 52). These findings collectively suggest that, although murine Irgm paralogs are involved in many processes that human IRGM is not, there exists significant functional homology pertaining to immunoregulation.

The dual roles of murine Irgm proteins in orchestrating protective cell-autonomous immunity to intracellular pathogens and regulating inflammation make it difficult to clearly appreciate their roles in shaping the pathophysiology of infections. Furthermore, the interregulatory relationships between the three Irgm proteins—exemplified by the reversal of lymphoid collapse and interferonopathy in *Irgm1*^−/−^ mice upon concurrent Irgm3 deletion among other phenotypes (33, 36, 37, 47)—obscure the individual roles of Irgm proteins and complicates the homology between mouse and human systems. For this reason, we leverage mice deficient for all three Irgm proteins (pan-*Irgm*^−/−^), revealing the collective function of all three Irgm proteins to best model a human system lacking its sole IRGM. Mirroring the finding that *IRGM* variants are associated with mortality in human *Mycobacterium tuberculosis* infection (40), pan-*Irgm*^−/−^ mice were recently shown to display worsened disease and mortality in *Mtb* infection (53). We employ genital *Chlamydia* infection in pan-*Irgm*^−/−^ mice as a model to evaluate protective immunity to this intracellular bacterial pathogen as well as regulation of pathogenic inflammation. We begin by demonstrating that, like *Irgm1*/*m3*^−/−^ mice, pan-*Irgm*^−/−^ mice are defective for cell-autonomous immunity to *C. trachomatis* (serovar L2) which corresponds with an early increase in bacterial burden *in vivo*. Compared to wild-type mice, pan-*Irgm*^−/−^ mice displayed extensively increased inflammation at 6 days post-infection (dpi). To uncouple the increase in bacterial burden from the increase in inflammation, we turned to the mouse-adapted *C. muridarum*, which evades IRG-mediated cell-autonomous immunity (8, 9). As expected, we observe no difference in *C. muridarum* burden between wild-type and pan-*Irgm*^−/−^ mice *in vitro* or *in vivo*, yet pan-*Irgm*^−/−^ mice display increased histologic inflammation at 6 dpi and 45 dpi as well as increased scarring pathology at 25 dpi and 45 dpi. We find that Irgm3 plays a more important role than Irgm1 or Irgm2 in controlling genital inflammation, and we show that excessive genital inflammation in pan-*Irgm*^−/−^ mice occurs independent of adaptive immunity. These findings demonstrate that Irgm proteins—especially Irgm3—regulate inflammation and immunopathology in genital *Chlamydia* infection independent of bacterial burden and establish genital *Chlamydia* infection as a useful model to study Irgm-regulated inflammation in the context of an infection.

## RESULTS

### Pan-*Irgm*^−/−^ mice are defective for cell-autonomous immunity to *C. trachomatis*

**Fig 1:**
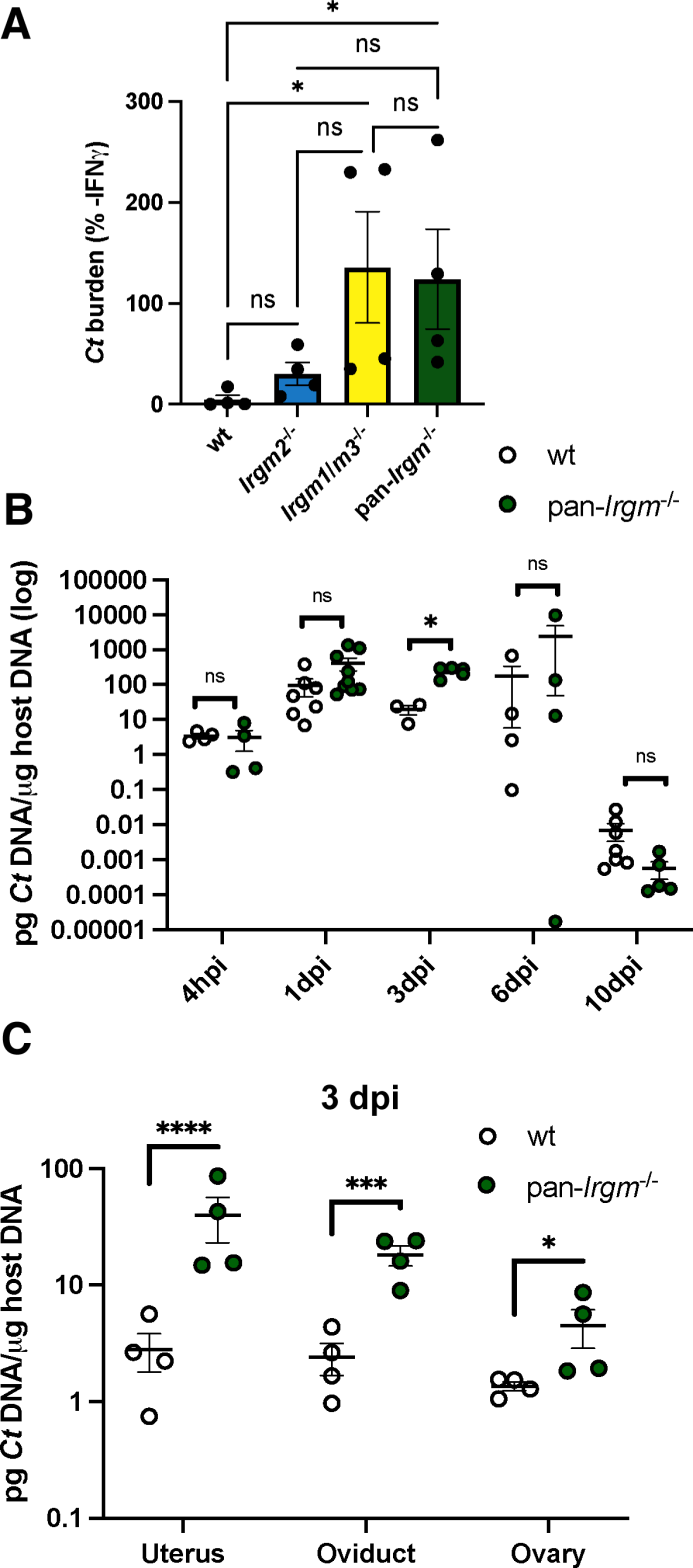
A defect in cell-autonomous immunity correlates with a transient increase in *C. trachomatis* burden in the female genital tract of pan-*Irgm*^−/−^ mice. (**A**) MEFs were primed overnight with 100 U/mL IFNγ prior to infection with *C. trachomatis* elementary bodies (EBs) at a multiplicity of infection (MOI) of 1:1. DNA was harvested from the infected cells at 24 hpi and bacterial burden was quantified using quantitative real-time polymerase chain reaction (qPCR). Graphs represent the proportion of bacterial burden in IFNγ-primed vs. unprimed cells. Data points represent MEFs derived from separate embryos (*n* = 4 separate MEF lines). Statistical significance was determined using one-way analysis of variance (ANOVA). (**B**) Mice were infected transcervically with 5 × 10^6^ *C. trachomatis* EBs, and female genital tracts were harvested at the indicated timepoints or (**C**) harvested at 3 dpi and then segmented. DNA was extracted from the harvested organ, and bacterial burden was quantified using qPCR. Each data point represents one mouse. Data in **B** represent pooled data from one experiment including all timepoints and one additional experiment for 3 dpi and 10 dpi timepoints: 4 hpi *n* = 4 mice; 1 dpi wild-type *n* = 7, pan-*Irgm*^−/−^ *n* = 9; 3 dpi wild-type *n* = 3, pan-*Irgm*^−/−^ *n* = 5; 6 dpi *n* = 4; and 10 dpi wild-type *n* = 7, pan-*Irgm*^−/−^ *n* = 5. Data in **C** represent one experiment (*n* = 4 mice). Statistical significance was determined using *t*-test (**B, C**); *P < 0.05, ***P < 0.0005, and ****P < 0.00005; ns, not significant.

We evaluated protective immunity to *C. trachomatis* in pan-*Irgm*^−/−^ mice and mice deficient for Irgm2 or doubly deficient for Irgm1 and Irgm3 (*Irgm1*/*m3*^−/−^); all mice were generated on a C57BL/6 background. While IFNγ-primed wild-type mouse embryonic fibroblasts (MEFs) robustly restrict the growth of *C. trachomatis*, it has been shown that *Irgm1*/*m3*^−/−^ MEFs display no IFNγ-mediated restriction of *C. trachomatis* growth, demonstrating a complete defect in cell-autonomous immunity to this bacterial pathogen (9, 37). The role for Irgm2 in cell-autonomous immunity to *C. trachomatis* has not been studied, but we and others have shown that Irgm2 promotes cell-autonomous immunity to *Toxoplasma* (38, 39). We have also shown that pan-*Irgm*^−/−^ mice are similarly defective for cell-autonomous immunity to *Toxoplasma* (39). We sought to determine if Irgm2 plays a similar role in cell-autonomous immunity to *C. trachomatis*. We found that while pan-*Irgm*^−/−^ MEFs displayed no IFNγ-mediated restriction of *C. trachomatis* growth, *Irgm2*^−/−^ MEFs restricted *C. trachomatis* growth similar to wild-type (Fig. 1A). This finding demonstrates that Irgm2 is largely dispensable for cell-autonomous immunity to *C. trachomatis* and pan-*Irgm*^−/−^ MEFs are completely defective for IFNγ-mediated anti-*C*. *trachomatis* host resistance, similar to *Irgm1*/*m3*^−/−^ MEFs.

While *Irgm1*/*m3*^−/−^ MEFs are completely defective for cell-autonomous immunity to *C. trachomatis*, *Irgm1*/*m3*^−/−^ mice demonstrate an early transient increase in bacterial burden *in vivo* but ultimately clear the infection with kinetics similar to wild-type mice (37). We next sought to determine the susceptibility of pan-*Irgm*^−/−^ mice to *in vivo* genital *C. trachomatis* infection. We infected mice transcervically with *C. trachomatis* and tracked bacterial burden over time by qPCR. Similar to previous reports in *Irgm1*/*m3*^−/−^ mice (37), pan-*Irgm*^−/−^ mice displayed increased bacterial burden relative to wild-type mice early in infection at 3 dpi, but bacterial burden is comparable to that of wild-type mice at subsequent timepoints (Fig. 1B). At 3 dpi, we observed an increase in *C. trachomatis* burden in the uteri, oviducts, and ovaries of infected pan-*Irgm*^−/−^ mice compared with the wild type (Fig. 1C). Therefore pan-*Irgm*^−/−^ mice display increased early bacterial burden *in vivo* that correlates with the expected *in vivo* timing of the observed *in vitro* defect in cell-autonomous restriction of bacterial growth, but additional immune mechanisms appear to ultimately compensate for this defect to restore protective immunity as in wild-type mice.

### *C. trachomatis*-infected pan-*Irgm*^−/−^ mice display increased genital inflammation and frequent granuloma formation

**Fig 2:**
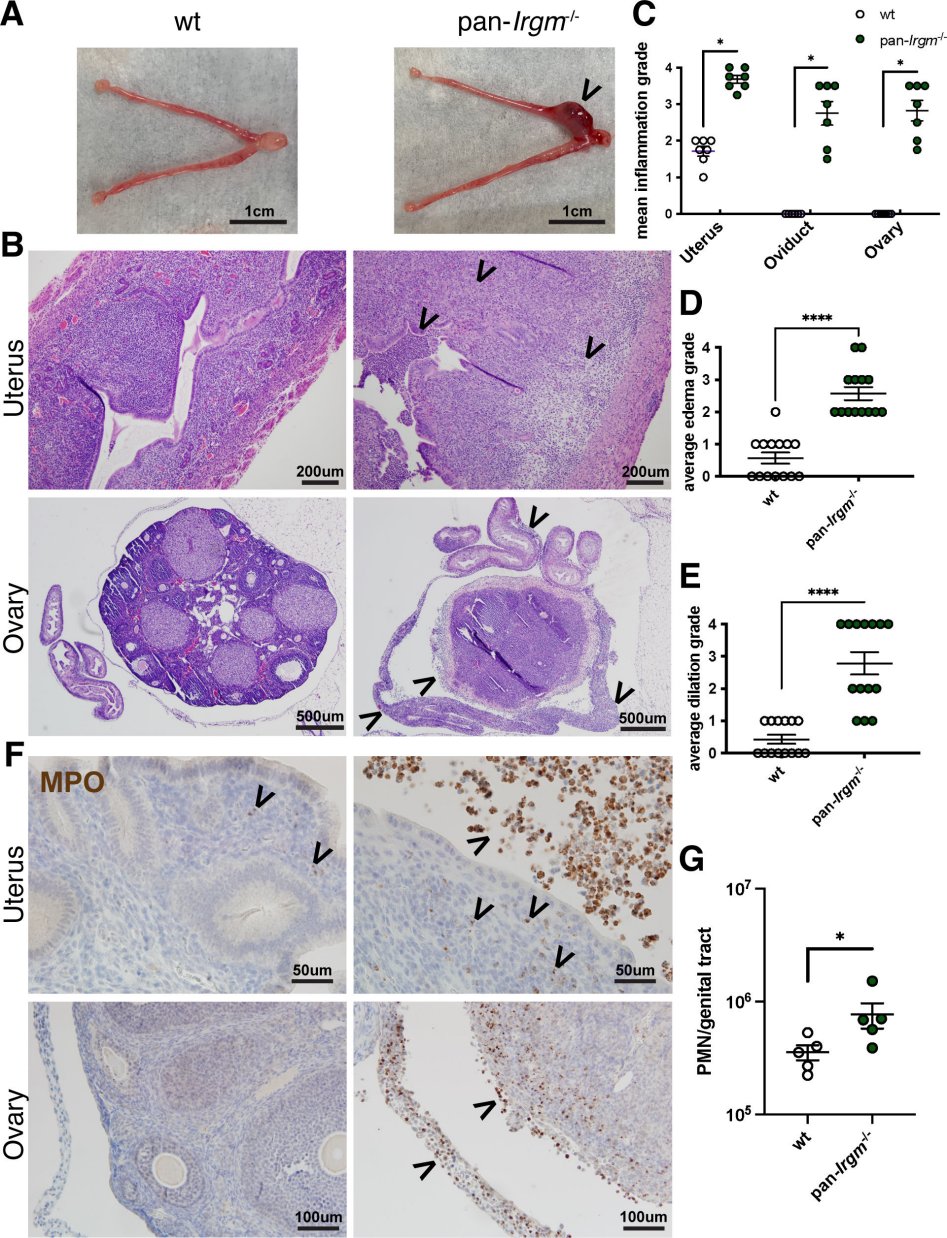
Pan-*Irgm*^−/−^ mice display increased inflammation in genital *C. trachomatis* infection. (**A–G**) Mice were infected transcervically with 5 × 10^6^ *C. trachomatis* EBs, and the female genital tract was harvested at 6 dpi. (**A**) Gross pathology—arrowhead indicates area of gross inflammation. (**B**) Genital tracts were fixed, sectioned, and H&E stained. Mildly inflamed wild-type uterine tissue and severely inflamed and edematous pan-*Irgm*^−/−^ uterus; wild-type ovary and oviduct with no evidence of inflammation and pan-*Irgm*^−/−^ ovary, oviduct, and mesosalpinx each displaying severe inflammation—arrowheads indicate regions of inflammatory infiltrate or edema. The magnitude of acute and chronic inflammation (**C**), edema (**D**), and dilation (**E**) was graded by a veterinary pathologist (*n* = 7 mice; each data point represents one uterine horn of an infected mouse). (**F**) Immunohistochemistry was performed on fixed genital tracts infected with *C. trachomatis* to label the neutrophil marker myeloperoxidase (MPO—brown). Arrowheads indicate regions or punctae of neutrophilic infiltrate. (**G**) Flow cytometry on genital tracts infected with *C. trachomatis* to quantify neutrophils (CD45+Gr1+ live cells; *n* = 5 mice; each data point represents one entire reproductive tract of an infected mouse). Data represent one experiment that is representative of multiple replicates. Statistical significance was determined using Mann-Whitney test (**C–E**) or *t*-test (**G**); *P < 0.05 and ****P < 0.00005.

Recent studies have implicated Irgm1 and Irgm2 in the control of inflammation in a variety of disease models (24, 43, 47, 51), but the collective roles of Irgm proteins in regulation of inflammation have only been described in macrophage responses to bacterial LPS and in *in vivo Mycobacterium tuberculosis* infection (52, 53). Because *Chlamydia* infection mediates disease predominantly via host inflammatory responses (6), we sought to use genital *Chlamydia* infection as a model to examine inflammation in pan-*Irgm*^−/−^ mice. We began by examining genital inflammation at 6 dpi in mice infected with *C. trachomatis* serovar L2, which causes minimal inflammation and pathology in wild-type mice (54). Consistent with prior reports, at this timepoint, wild-type mice displayed mild inflammation in the uterus and no inflammation in the oviducts and ovaries. Pan-*Irgm*^−/−^ mice, in contrast, displayed increased uterine inflammation and tissue destruction, as well as significant inflammation in the oviducts and ovaries (Fig. 2A through E). Additionally, there was increased neutrophil influx in pan-*Irgm*^−/−^ genital tracts compared with the wild type (Fig. 2F and G).

**Fig 3:**
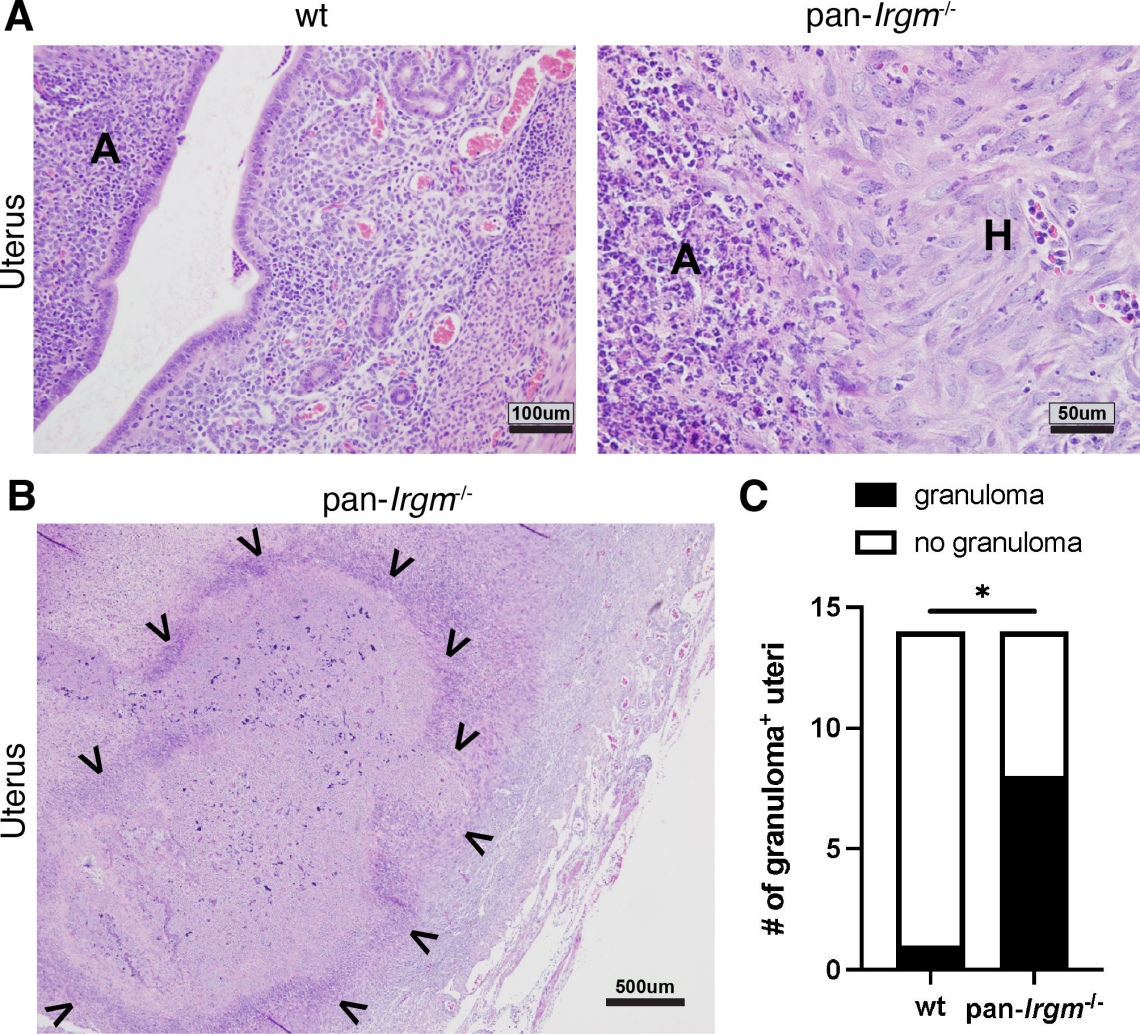
Pan-*Irgm*^−/−^ mice display granulomatous inflammation in genital *C. trachomatis* infection. Mice were infected transcervically with 5 × 10^6^ *C. trachomatis* EBs and harvested at 6 dpi. (**A**) High magnification of mild acute inflammation in wild-type uterus and acute and histiocytic inflammation in pan-*Irgm*^−/−^ uterus. A, acute inflammation; H, histiocytic inflammation. (**B**) Low magnification of diffuse granulomatous inflammation in pan-*Irgm*^−/−^ uterus. Arrowheads indicate margins of granulomatous lesion. (**C**) Quantification of number of uterine horns displaying granulomatous lesions (*n* = 7 mice). Data represent one experiment that is representative of multiple replicates. Statistical significance was calculated using χ^2^ test; *P < 0.05.

Noticeably, uteri of pan-*Irgm*^−/−^ mice displayed not only signs of acute but also histiocytic (Fig. 3A) as well as granulomatous inflammation (Fig. 3B). While uterine granulomas rarely formed in wild-type mice (1 out of 14), granulomas were found in the uteri of more than half of infected pan-*Irgm*^−/−^ mice (8 out of 14; Fig. 3C). Collectively, the stark increase in inflammation and tissue destruction in pan-*Irgm*^−/−^ mice suggests that Irgm proteins regulate genital inflammation in *C. trachomatis* infection, potentially in addition to their roles in host resistance.

### Irgm proteins do not promote defense against *C. muridarum* infection

**Fig 4:**
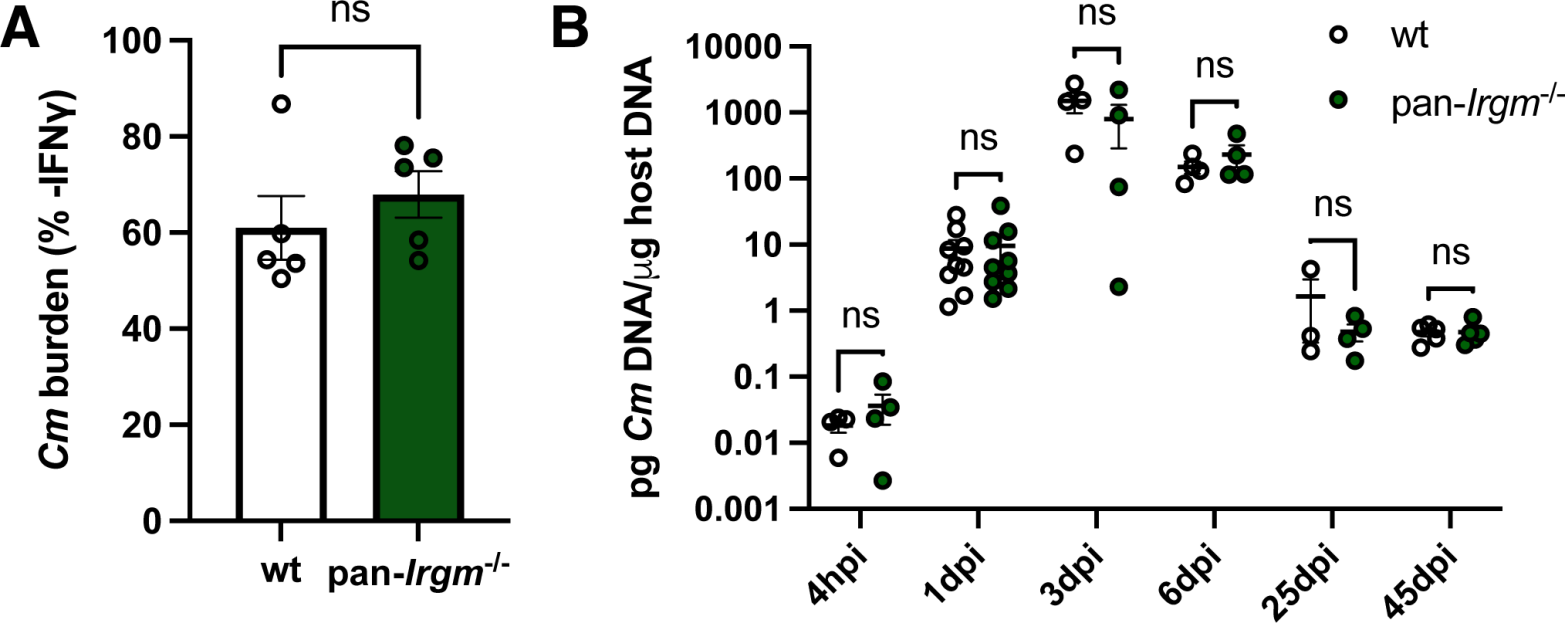
Host resistance to *C. muridarum* remains unchanged in the absence of all three murine Irgm paralogs. (**A**) MEFs were primed overnight with IFNγ prior to infection with *C. muridarum* EBs at an MOI of 1:1. DNA was harvested at 24 hpi, and bacterial burden was quantified using qPCR. Graphs represent the proportion of bacterial burden in IFNγ-primed vs. unprimed cells. Data points represent distinct MEF lines derived from separate embryos (*n* = 5 separate MEF lines). (**B**) Mice were infected transcervically with 2.5 × 10^5^ *C. muridarum* EBs, and genital tracts were harvested at the indicated timepoints. DNA was extracted and bacterial burden was quantified using qPCR. Data represent pooled data from one experiment including all timepoints plus one additional experiment for the 1-dpi timepoint: 4 hpi *n* = 4 mice; 1 dpi *n* = 9; 3 dpi *n* = 4; 6 dpi *n* = 4; 25 dpi wild-type *n* = 3, pan-*Irgm*^−/−^ *n* = 4; and 45 dpi *n* = 5. Statistical significance was determined using *t*-test; ns, not significant.

Pathogen load is a major factor that drives inflammation. While *C. trachomatis* burden was similar between wild-type and pan-*Irgm*^−/−^ mice at 6 dpi and 10 dpi, there was increased bacterial load at 3 dpi throughout the genital tracts of pan-*Irgm*^−/−^ mice (Fig. 1). Therefore, Irgm proteins could limit inflammation of the infected female genital tract in part or entirely by promoting cell-autonomous immunity and restriction of bacterial growth. Alternatively, the exacerbation of the inflammatory response seen in pan-*Irgm*^−/−^ mice could be explained if Irgm proteins additionally attenuated inflammation independent of bacterial burden in chlamydial infection. To be able to test the latter hypothesis, we leveraged the mouse-adapted strain *C. muridarum*, which evolved to evade IFNγ-mediated cell-autonomous immunity executed by the family of murine IRG proteins (8–10, 32). Confirming that *C. muridarum* is resistant to Irgm-mediated cell-autonomous immunity, we found that both wild-type and pan-*Irgm*^−/−^ MEFs displayed similarly limited IFNγ-mediated restriction of *C. muridarum* growth (Fig. 4A). We next infected wild-type and pan-*Irgm*^−/−^ mice transcervically with *C. muridarum* and tracked bacterial burden *in vivo* over time using qPCR. In contrast to *C. trachomatis* (Fig. 1), we found no difference in *C. muridarum* burden in wild-type and pan-*Irgm*^−/−^ mice at any timepoint (Fig. 4B). These findings demonstrate that Irgm proteins do not play meaningful roles in the clearance of *C. muridarum* in the female genital tract and provided us with an experimental platform to probe for anti-inflammatory function of Irgm proteins independent of their role as host resistance factors.

### Pan-*Irgm*^−/−^ mice display increased inflammation and immunopathology in *C. muridarum* infection

**Fig 5:**
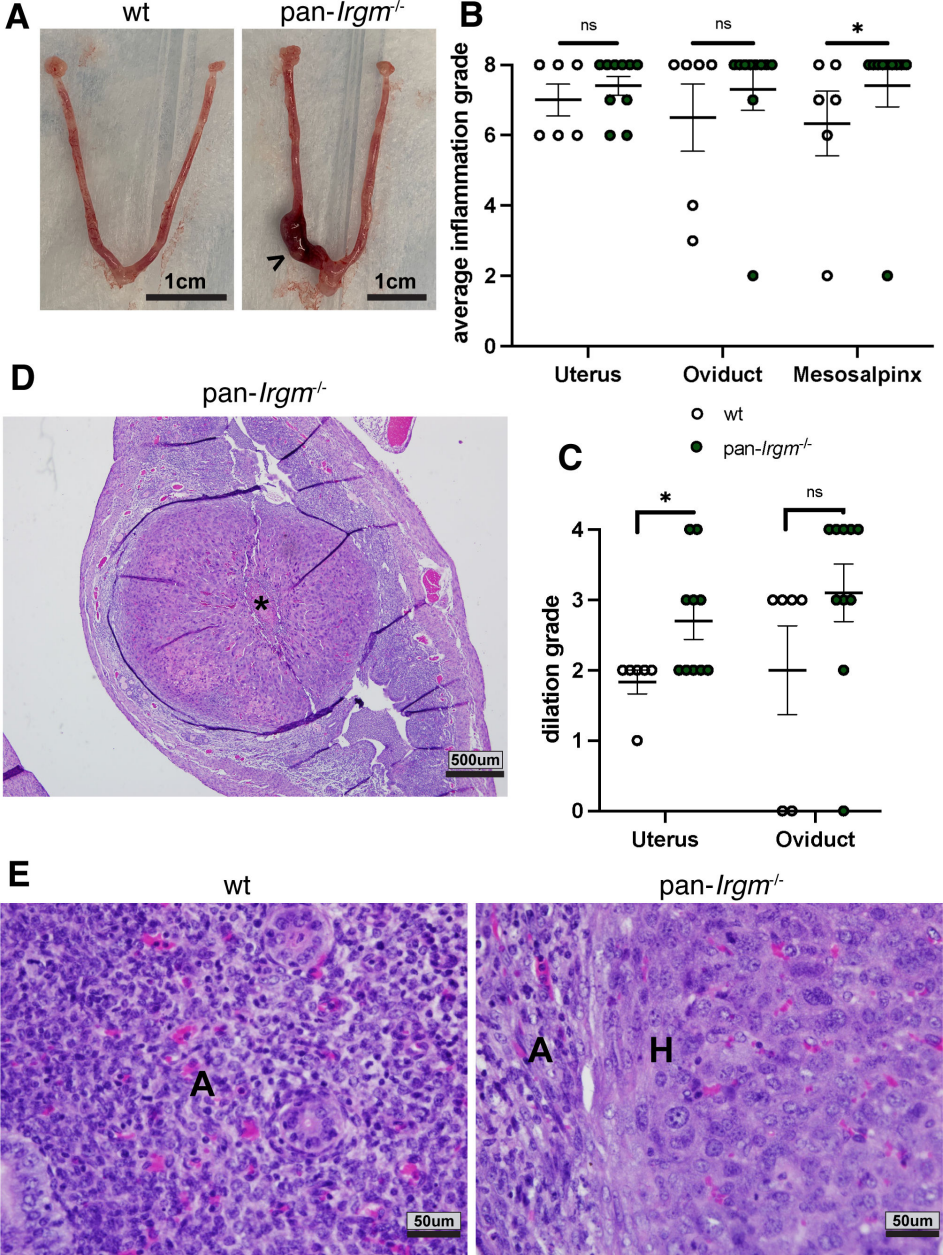
Increased inflammation in pan-*Irgm*^−/−^ at 6 dpi in genital *C. muridarum* infection. Mice were infected transcervically with 2.5 × 10^5^ *C. muridarum* EBs and harvested at 6 dpi (**A–E**). (**A**) Gross pathology of infected genital tracts. Arrowhead denotes region of gross inflammation. (**B–E**) Genital tracts were fixed, sectioned, and H&E stained. The magnitude of acute and chronic inflammation (**B**) and dilation (**C**) was graded by a veterinary pathologist. (**D**) Low magnification of granulomatous lesion (*) in pan-*Irgm*^−/−^ uterus. (**E**) High magnification of acute inflammation in wild-type uterus and acute and histiocytic inflammation in pan-*Irgm*^−/−^ uterus. A, acute inflammation; H, histiocytic inflammation. Data represent one experiment that is representative of multiple replicates. Wild-type *n* = 6 mice, pan-*Irgm*^−/−^ *n* = 10; each data point represents one uterine horn of an infected mouse. Statistical significance was determined using Mann-Whitney test; *P < 0.05, ns = not significant.

Because wild-type and pan-*Irgm*^−/−^ mice displayed indistinguishable levels of bacterial burden in genital *C. muridarum* infection, any change in inflammation in pan-*Irgm*^−/−^ mice would necessarily be attributable to the roles for Irgm proteins in the regulation of inflammation. We therefore began by examining inflammation at 6 dpi in transcervical *C. muridarum* infection. In contrast to *C. trachomatis* L2 infection, *C. muridarum* induced significant inflammation and tissue destruction in both wild-type and pan-*Irgm*^−/−^ mice throughout the genital tract at 6 dpi. In every measure, pan-*Irgm*^−/−^ mice displayed slightly increased inflammation and markers of tissue destruction, and this difference reached statistical significance for uterine dilation and mesosalpingeal inflammation (Fig. 5A through C). Pan-*Irgm*^−/−^ mice infected with *C. muridarum* also displayed large granulomatous lesions (Fig. 5D) and histiocytic inflammation compared with acute, neutrophilic inflammation in wild-type mice (Fig. 5E). While the severity of inflammation already observed in wild-type genital tracts infected with *C. muridarum* makes it difficult to appreciate increases in inflammation in pan-*Irgm*^−/−^ mice, the nonetheless statistically significant increases in pathology scores indicate that Irgm proteins dampen inflammation in *C. muridarum* infection at 6 dpi independent of bacterial burden.

**Fig 6:**
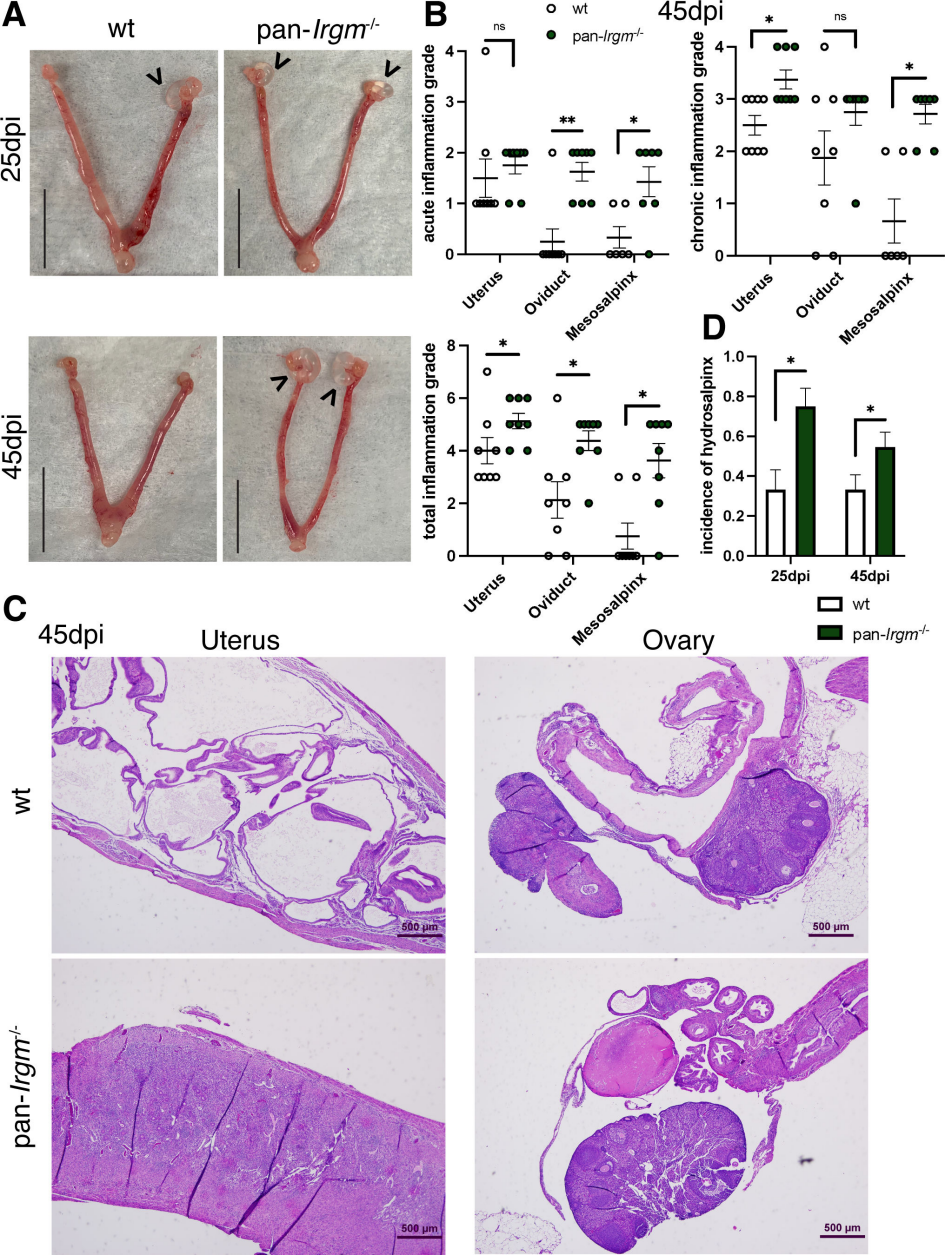
Increased pathology in pan-*Irgm*^−/−^ mice at later timepoints in genital *C. muridarum* infection. Mice were infected transcervically with 2.5 × 10^5^ *C. muridarum* EBs and harvested at 25 dpi or 45 dpi. (**A**) Gross pathology of infected genital tracts. Scale bars = 0.5 in; arrowheads indicate hydrosalpinx. (**B–D**) Genital tracts were fixed, sectioned, and H&E stained. (**B**) The magnitude of acute, chronic, and total acute + chronic inflammation at 45 dpi was graded by a veterinary pathologist (wild-type *n* = 7 mice, pan-*Irgm*^−/−^ *n* = 8). Data represent one experiment that is representative of multiple replicates. (**C**) Low-magnification images of cystic changes in wild-type uterus and ovary/oviduct and continued inflammation in pan-*Irgm*^−/−^ uterus and ovary/oviduct. (**D**) Quantification of hydrosalpinx at 25 dpi and 45 dpi in genital tracts infected with *C. muridarum*. Data represent pooled data from three experiments (25 dpi *n* = 12 mice; 45 dpi wild-type *n* = 21, pan-*Irgm*^−/−^ *n* = 22). Each data point represent one uterine horn of an infected mouse. Statistical significance was determined using Mann-Whitney tests (**B**) or χ^2^ test (**D**); *P < 0.05 and **P < 0.005; ns, not significant.

We next examined inflammation at 45 dpi (Fig. 6A), when bacterial burden was reduced substantially compared with 6 dpi (Fig. 4B). At 45 dpi, there was increased inflammation throughout the genital tracts of pan-*Irgm*^−/−^ mice infected with *C. muridarum* compared with the wild type. By 45 dpi, there was very little neutrophilic infiltration in the oviducts and mesosalpinges of wild-type mice; however, there was still moderate neutrophil infiltration in the oviducts and mesosalpinges of pan-*Irgm*^−/−^ mice. Chronic inflammation characterized by lymphocytic infiltration continued in wild-type genital tracts, although to a more significant degree in pan-*Irgm*^−/−^ uteri and mesosalpinges (Fig. 6B). Histologically, wild-type mice displayed cystic changes with minimal foci of inflammation, while pan-*Irgm*^−/−^ mice displayed more severe and widespread inflammation that included the oviducts and ovaries (Fig. 6C). These findings demonstrate that pan-*Irgm*^−/−^ mice display continued acute inflammation and increased chronic inflammation in *C. muridarum* infection at late timepoints.

*Chlamydia* causes genital inflammation that results in tissue scarring and pathology in mouse models (6). We therefore sought to examine the incidence of hydrosalpinx, a reliable marker of *Chlamydia*-related oviduct scarring, in wild-type and pan-*Irgm*^−/−^ mice infected with *C. muridarum* at additional timepoints. We found that there was a substantial increase in the incidence of hydrosalpinx at 25 dpi and 45 dpi in pan-*Irgm*^−/−^ mice compared with wild-type mice (Fig. 6A and D). This finding demonstrates that Irgm proteins limit scarring pathology and continued inflammation in genital *C. muridarum* infection and thus define Irgm proteins as mediators of disease tolerance to chlamydial infections.

### Irgm3 is the most critical Irgm paralog in controlling genital inflammation in *C. muridarum* infection

**Fig 7:**
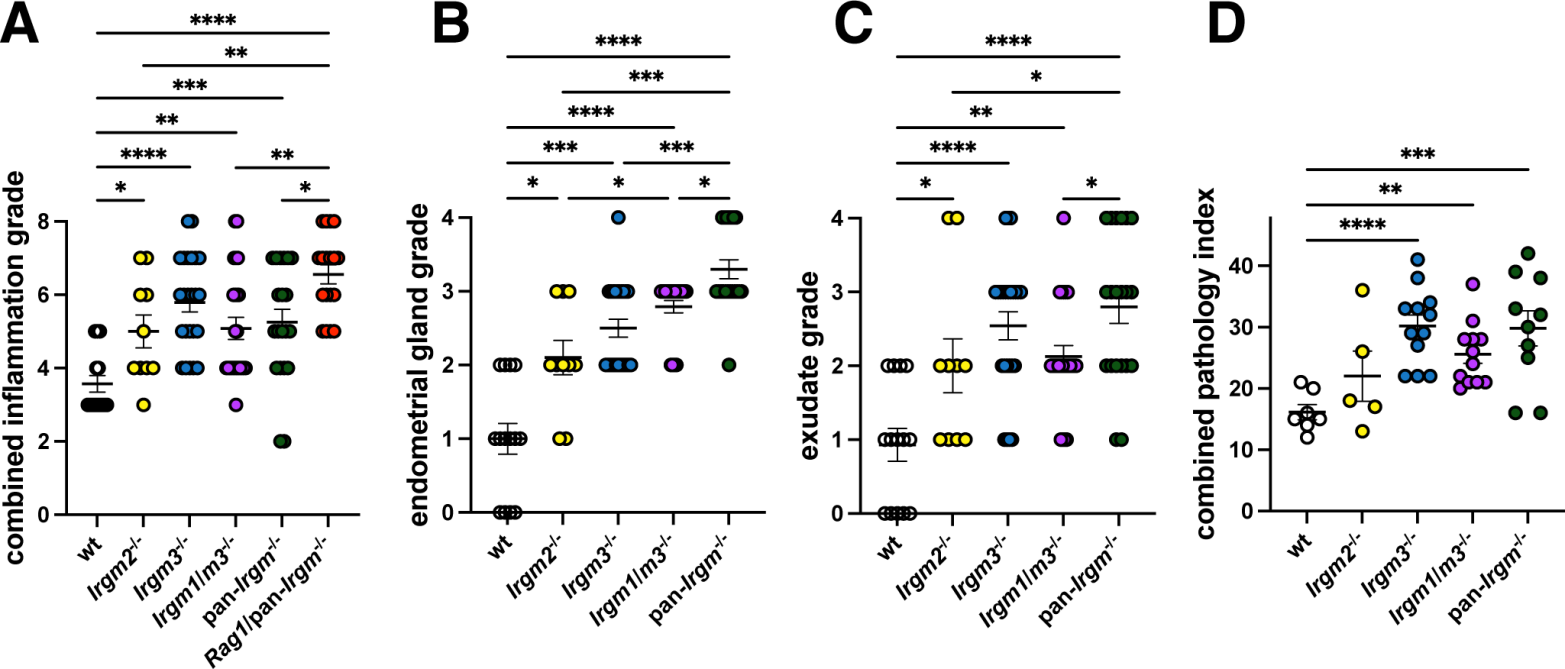
Increased inflammatory pathology in Irgm- and Rag1-deficient uteri in genital *C. muridarum* infection at 45 dpi. Mice were infected transcervically with 2.5 × 10^5^ *C. muridarum* EBs. Genital tracts were harvested at 45 dpi, fixed, sectioned, H&E stained, and graded by a veterinary pathologist. (**A**) The magnitude of acute and chronic inflammation in the uteri as defined by standard scoring criteria was combined. Additional features such as endometrial glandular pathology (**B**) and uterine luminal exudates (**C**) were also included. (A –C) Each data point represents one uterine horn of an infected mouse. (**D**) A combined uterine pathology index consisting of the sum of acute inflammation, chronic inflammation, dilation, glandular pathology, luminal exudates, and granulomatous inflammation was calculated. Each data point represents combined pathology scores from one entire reproductive tract from an infected mouse. Figure represents pooled data from two independent experiments (wild-type *n* = 7 mice, *Irgm2*^−/−^ *n* = 5, *Irgm3*^−/−^ *n* = 12, *Irgm1*/*m3*^−/−^ *n* = 12, pan-*Irgm*^−/−^ *n* = 10, and *Rag1*/pan*-Irgm*^−/−^ *n* = 9). Statistical significance was determined using Mann-Whitney tests; *P < 0.05, **P < 0.005, ***P < 0.0005, and ****P < 0.00005.

Next, we asked whether these effects were driven by one or a combination of Irgm paralogs. Because *Irgm1*^−/−^ mice develop spontaneous and often severe interferonopathy as a confounding factor, we instead opted to test Irgm1 function in mice in which type I interferon response had been normalized through co-deletion of Irgm3 (47). In addition to these *Irgm1*/*m3*^−/−^ mice, we also infected *Irgm2*^−/−^ and *Irgm3*^−/−^ mice transcervically with *C. muridarum* alongside wild-type and pan-*Irgm*^−/−^ mice and histologically examined inflammation and inflammatory pathology at 45 dpi. All Irgm-deficient phenotypes demonstrated increased combined acute and chronic inflammation in the uterus of infected mice (Fig. 7A). In many standard metrics, such as acute inflammation alone, chronic inflammation alone, and tissue dilation, it was difficult to clearly separate different Irgm-deficient phenotypes from one another when examining pathology in the uterus, oviduct, and ovaries (Fig. S1A through C). We therefore designed additional pathologic scoring criteria to better characterize phenotypes that were observed but not captured in the standard pathology grading criteria. Grading infected uteri for endometrial gland infiltration and dilation (Fig. 7B), as well as uterine luminal exudate (Fig. 7C), clearly revealed these as pathologic features in Irgm-deficient mice, with *Irgm3*^−/−^ and pan-*Irgm*^−/−^ uteri largely presenting as more severe than *Irgm2*^−/−^ uteri. *Irgm1*/*m3*^−/−^ mice generally phenocopied *Irgm3*^−/−^ mice, indicating that Irgm1 is unlikely to impact the inflammatory response to *C. muridarum* in the female genital tract. Combining all pathological grading criteria into a combined pathology index revealed the same trend (Fig. 7D). These findings demonstrate that Irgm1 and Irgm2 play more minor roles and Irgm3 plays a more major role in limiting inflammation and tissue distortion and promoting disease tolerance in genital *C. muridarum* infection.

### Increased genital inflammation in pan-*Irgm*^−/−^ mice infected with *C. muridarum* is not dependent on adaptive immunity

Irgm proteins limit immune responses by a variety of different mechanisms in different contexts. Specifically, Irgm3 has been shown to regulate inflammation via regulating antigen presentation (55, 56), and Irgm1 and Irgm3 regulate T cell responses in genital *C. trachomatis* infection (37). Additionally, certain varieties of T cells mediate inflammatory pathology in genital *Chlamydia* infection (6). We sought to determine whether the increased inflammation observed in pan-*Irgm*^−/−^ mice was dependent on adaptive immunity. To this end, we generated mice deficient for all three Irgm proteins as well as Rag1, a protein critical for functional T and B cell development (*Rag1*/pan-*Irgm*^−/−^). We included these mice alongside wild-type, *Irgm2*^−/−^, *Irgm3*^−/−^, *Irgm1*/*m3*^−/−^, and pan-*Irgm*^−/−^ mice for transcervical *C. muridarum* infection and histological analysis of genital inflammation at 45 dpi. We found that *Rag1*/pan-*Irgm*^−/−^ mice displayed increased inflammation and inflammatory pathology in the uteri, oviducts, and ovaries across all applied metrics, compared with wild-type mice as well as all Irgm knockout models (Fig. 7A; Fig. S1). Because the magnitude of inflammation was radically greater in *Rag1*/pan-*Irgm*^−/−^ mice compared with all other groups, they were not included in the expanded grading criteria in Fig. 7B through D. These findings demonstrate that adaptive immune cells such as cytotoxic CD8 T cells are dispensable for the formation of the severe pathology observed in *C. muridarum*-infected pan-*Irgm*^−/−^ mice. Rather, our data indicate that adaptive immunity promotes disease tolerance in genital *C. muridarum* infection in Irgm-deficient mice.

## DISCUSSION

Irgm1 and Irgm3 have been shown to be critical regulatory factors for IFNγ-mediated defense against *C. trachomatis*, but the collective roles of Irgm proteins in host defense and the regulation of inflammation in *Chlamydia* infection have not been studied. We report that pan-*Irgm*^−/−^ MEFs are completely deficient for IFNγ-mediated resistance to *C. trachomatis*, correlating with a moderate transient defect in resistance *in vivo* and a disproportional increase in genital inflammation at 6 dpi. We show that pan-*Irgm*^−/−^ and wild-type MEFs and mice are equally resistant to *C. muridarum* infection, yet pan-*Irgm*^−/−^ mice display an increase in histologic inflammation at 6 dpi and 45 dpi and an increase in hydrosalpinx at 25 dpi and 45 dpi. We found that these phenotypes were driven more by a deficiency in Irgm3 compared with Irgm2 and Irgm1. These findings demonstrate that Irgm proteins confer resistance to disease caused by *C. trachomatis—*a pathogen that is susceptible to murine mechanisms of cell-autonomous immunity—and also confer relative disease tolerance to infections with mouse-adapted *C. muridarum* by controlling destructive inflammatory responses. Genital *Chlamydia* infection in pan-*Irgm*^−/−^ mice is therefore an effective infection model to study the roles of Irgm proteins in both disease resistance and disease tolerance.

The phenotypic differences between infection with *C. trachomatis* and *C. muridarum* are consistent with existing models of host tropism and susceptibilities to murine immunity. Innate immunity in mice with a functional IRG system has been shown to be sufficient to clear *C. trachomatis* genital infection (57). The early increase in *C. trachomatis* burden *in vivo* correlates well with our finding that pan-*Irgm*^−/−^ MEFs lack IFNγ-induced cell-autonomous immunity *in vitro*, which is consistent with a defect in epithelial cells and other non-immune cells to restrict bacterial burden *in vivo* prior to activation of professional immune cells. Macrophages and neutrophils, which have been shown to kill *Chlamydia in vitro*, possess additional antimicrobial mechanisms beyond the IRG system including the generation of radical oxygen species (58–61). Robust macrophage and neutrophil responses after 3 dpi, followed by engagement of adaptive immunity after the first week of infection, correlate with the decrease in *C. trachomatis* burden after 3 dpi and ultimate clearance of infection. In contrast, *C. muridarum* has adapted to evade cell-autonomous immunity in its preferred rodent host. Because murine cell-autonomous immunity to *Chlamydia* hinges upon the IRG system, pan-*Irgm*^−/−^ mice display wild-type-level resistance to *C. muridarum* infection both *in vitro* and *in vivo*. The susceptibilities *in vitro* and *in vivo* of pan-*Irgm*^−/−^ to infection with *C. trachomatis* and *C. muridarum* fit elegantly within known models for the host tropism of those two bacterial pathogens.

The increase in inflammation in pan-*Irgm*^−/−^ mice infected with *C. trachomatis* compared with the wild type was striking, and while it correlated with the early defect in control of bacterial growth, the magnitude of the increase in pathology appeared out of proportion with the increase in bacterial growth. While we hypothesize that the absence of immunoregulatory Irgm proteins contributes to the observed increase in inflammation, the increased bacterial burden makes it difficult to appreciate the roles for Irgm proteins in promoting disease tolerance in *C. trachomatis* infection. Genital *C. muridarum* infection thus provides a useful model in that bacterial burden is unchanged in pan-*Irgm*^−/−^ compared with wild-type mice. In this system, differences in inflammation and inflammatory pathology are indicative only of the effects of deletion of Irgm proteins on the regulation of inflammation.

Irgm proteins have been shown to regulate inflammation via multiple mechanisms, paralleling roles for human IRGM as demonstrated in cellular studies as well as disease phenotypes associated with genetic *IRGM* variants. Variants in human *IRGM* are associated with increased mortality in severe sepsis and increased incidence of inflammatory bowel disease and other autoimmune diseases, and the disease-associated variants are generally correlated with decreased IRGM expression (22, 62). Certain variants in *IRGM* are also associated with increased incidence of active pulmonary *Mycobacterium tuberculosis* disease (40). Because IRGM has been shown to facilitate resistance to *Mycobacterium tuberculosis in vitro* via phagosome-lysosome fusion (41), it is assumed that the increased incidence in mycobacterial disease associated with *IRGM* variants is caused by a defect in disease resistance. However, our findings in genital *Chlamydia* infection along with recent studies demonstrating that pan-*Irgm*^−/−^ mice display altered cytokine profiles and worsened mortality in *Mycobacterium tuberculosis* infection (53) highlight the possibility that a failure in human IRGM-mediated disease tolerance also contributes to the observed increase in mycobacterial disease in susceptible individuals.

*Irgm1*^−/−^ mice display numerous phenotypes that parallel these inflammatory associations of human *IRGM*, including an increased susceptibility to intestinal inflammation in multiple models, a Sjogren’s syndrome-like autoimmune phenotype underlined by a type I interferonopathy, and a defect in resistance to mycobacterial infection (41–44, 47, 63, 64). Mechanistically, both human IRGM and murine Irgm1 control canonical inflammasome activity via selective autophagy and have been shown to promote mitochondrial homeostasis (23, 25, 46, 65). In *Irgm1*^−/−^ cells, mitochondrial instability results in increased inflammatory signaling and type I interferon production (24, 47). Irgm1 also regulates lymphocyte and leukocyte homeostasis, although homologous functions have not been investigated in humans (37, 48, 49). Additionally, Irgm2 has been implicated in controlling noncanonical inflammasome activation upon stimulation with LPS or infection with *E. coli* (51, 52). Irgm3 contributes to the regulation of immune responses via antigen presentation (55, 56). These mechanisms have also not been investigated in human systems.

There is no known role for human IRGM in *Chlamydia* infection, and because of the vastly divergent mechanisms of cell-autonomous immunity between mice and humans, it is exceedingly unlikely that human IRGM regulates protective immunity to *C. trachomatis*, as supported by some experimental evidence (66). However, the parallels in immune phenotypes between mouse and human models of IRGM deficiency strongly support functional homology in immunoregulation across species. It is distinctly possible that human IRGM promotes disease tolerance in *Chlamydia* infection similar to murine Irgm proteins and that similar parallels may exist in infection with other pathogens such as *M. tuberculosis*. The mechanisms by which inflammation is dysregulated in pan-*Irgm*^−/−^ mice could therefore elucidate the roles for human IRGM in human disease.

Several specific immune mechanisms have been implicated in mediating pathology in genital *C. muridarum* infection, including neutrophils and CD8+ T cells as well as the cytokines IL-1 and TNFα (6). Our finding that pan-*Irgm*^−/−^ mice display increased hydrosalpinx in *C. muridarum* infection compared with the wild type indicates that Irgm proteins regulate pathogenic immune responses. For example, it is possible that pan-*Irgm*^−/−^ mice display increased inflammasome activation or increased signaling via another mechanism of bacterial sensing. It is also possible that Irgm proteins regulate lymphocyte or leukocyte homeostasis, resulting in increased neutrophil or CD8+ T cell activation in pan-*Irgm*^−/−^ mice. However, our finding that *Rag1*/pan-*Irgm*^−/−^ mice have worsened rather than improved pathology in *C. muridarum* infection compared with pan-*Irgm*^−/−^ mice demonstrates that adaptive immunity as a whole limits inflammation in these mice. However, deletion of Rag1 concurrently interferes with adaptive responses that are pathogenic (e.g., CD8+ T cells) and protective (e.g., IFNγ-secreting Th1 cells or suppressive regulatory T cells) in *C. muridarum* infection (6). Further experiments targeting more specific branches of adaptive immunity in Irgm-deficient cells will help shed light on these mechanisms.

The persistence of active acute and chronic inflammation in pan-*Irgm*^−/−^ mice infected with *C. muridarum* at 45 dpi despite no increase in bacterial burden throughout infection suggests that Irgm proteins are required for the control and resolution of inflammation. The observed increase in granulomatous inflammation in *Chlamydia*-infected pan-*Irgm*^−/−^ mice was particularly striking and intriguing, as Crohn’s disease, which is linked with polymorphisms in *IRGM*, is characterized by intestinal granulomatous inflammation (67). Increased and persistent granulomatous inflammation could imply defects in the clearance of bacterial antigens, dysregulation of macrophage activity or signaling, or a failure to control lymphocyte activity. While all three Irgm paralogs appear to play distinct roles in controlling immunity to genital *C. muridarum* infection, our observation that Irgm3-deficient mice displayed essentially the same phenotypic features as pan-*Irgm*^−/−^ mice (including granulomatous inflammation) suggests that future studies could be directed specifically at Irgm3. Our findings address the collective roles of Irgm proteins in regulating inflammation to promote disease tolerance, establishing genital *Chlamydia* infection in pan-*Irgm*^−/−^ mice as an effective model to study these phenotypes, and implicating Irgm proteins in specific inflammatory processes that may suggest parallel roles for human IRGM.

## MATERIALS AND METHODS

### Cell lines and bacterial strains

Primary MEFs were derived from wild-type C57BL/6, *Irgm2*^−/−^, *Irgm1*/*m3*^−/−^ and pan-*Irgm*^−/−^ mice as described previously (52). Briefly, the head and vital organs were removed from E12.5–E14.5 embryos, embryos were minced and digested in 0.25% trypsin (Gibco) at 37C. MEFs and African green monkey kidney Vero cells were cultured in Dulbecco’s modified Eagle medium supplemented with 10% heat-inactivated fetal bovine serum, penicillin (100 U/mL), and streptomycin (100 µg/mL). Cells to be used for *Chlamydia* infection were cultured without antibiotics.

*Chlamydia trachomatis* serovar L2 expressing GFP and the *C. muridarum* AR Nigg strain (68) were propagated in Vero cells, and EBs were purified as described (32).

### Quantitative real-time polymerase chain reaction

DNA was extracted from cells and tissue using the DNeasy Blood and Tissue DNA extraction kit (Qiagen). Tissue was harvested from infected mice and homogenized using a Power Gen 500 tissue homogenizer (Fisher Scientific) prior to DNA extraction. For quantification of DNA, samples were run in duplicate using TaqMan Fast Advanced Master Mix (Thermo Fisher) according to the manufacturer’s protocol. For quantification of host DNA, primers and fluorescent probes included in the TaqMan Rodent GAPDH Control Reagents kit (Thermo Fisher) were used. For quantification of *Chlamydia* DNA, custom primers and probes designed to amplify and label the *Chlamydia* 16S gene were used (forward: 5′-GGAGGCTGCAGTCGAGAATCT-3′; reverse: 5′-TTACAACCCTAGAGCCTTCATCACA-3′; probe 5′-6FAM-TCGTCAGACTTCCGTCCATTGCGA-TAM-3′; Fisher Scientific/Eurofins).

### Measurement of *in vitro Chlamydia* burden

For quantification of *Chlamydia* burden *in vitro*, primary MEFs were incubated with or without 100 U/mL IFNγ overnight prior to synchronous infection with *Chlamydia* EBs by centrifugation at 4°C at an MOI of 1:1. Cells were harvested 24 hpi for DNA extraction and quantitative real-time polymerase chain reaction (qPCR). *Chlamydia* and host DNA mass were quantified using a standard curve generated using purified host and *Chlamydia* DNA and the same qPCR reagents used throughout each experiment. Bacterial burden in IFNγ-primed cells was divided by the bacterial burden in the corresponding unprimed cells of the same genotype. Graphs represent pooled data from at least three independent experiments conducted using MEFs derived from different embryos.

### Mouse husbandry and *in vivo* genital *Chlamydia* infection

All mouse lines were maintained at animal facilities at Duke University Medical Center. Animal protocols were approved by the Institutional Animal Care and Use Committees at Duke University. Wild-type C57BL/6 (Jackson Laboratories), *Irgm2*^−/−^, pan-*Irgm*^−/−^ (52), *Irgm3*^−/−^, *Irgm1*/*m3*^−/−^ (33), and *Rag1*/pan-*Irgm*^−/−^ mice were used. All knockout mouse lines are on a C57BL/6 background as described in the relevant citations. For all experiments, 6–12-week-old mice were injected subcutaneously with 2.5 mg medroxyprogesterone acetate (Depo-Provera, Pfizer) diluted in sterile PBS 1 week prior to infection. *Chlamydia* EBs were diluted in sucrose-phosphate-glutamate (SPG) buffer (10 mM sodium phosphate, 220 mM sucrose, and 0.50 mM L-glutamic acid). For transcervical infection, 5 µL of diluted *Chlamydia* was instilled directly into the uterus using a Non-Surgical Embryo Transfer device (Braintree Scientific). For *in vivo C. trachomatis* infection, 5 × 10^6^ IFU was delivered per mouse, and for *in vivo C. muridarum* infection, 2.5 × 10^5^ IFU was delivered per mouse. For all mock infections, sterile SPG was used. Infected mice were monitored over the course of each infection.

### Evaluation of genital pathology

Genital tracts were removed surgically, and pictures of gross pathology were taken using an iPhone XR. Hydrosalpinx was evaluated visually upon removal of the genital tract. Genital tracts were fixed in 10% formalin before routine processing to paraffin, microtomy at 5 µm, and hematoxylin and eosin staining. Semiquantitative grading of the severity of pathological features was performed by a board-certified veterinary anatomic pathologist (JE) as described (69). Acute inflammation, chronic inflammation, edema, dilation, and fibrosis were graded on the following scale from 0 to 4: 0 = normal; 1 = rare foci of inflammation; 2 = scattered (1–4) aggregates of inflammation or mild diffuse inflammation; 3 = numerous aggregates (>4) of inflammation or moderate diffuse inflammation; and 4 = severe diffuse infiltration or confluence of inflammation. In Fig. 7 and Fig. S1, additional grading criteria were used to assess endometrial glandular inflammation, endometrial exudates, and granulomatous lesions. Left and right genital tracts were graded separately. Scoring was performed in a masked fashion without knowledge of allocation group assignment. Graphs represent the sums of acute and chronic inflammation scores in the left and right sides of each genital tract.

### Statistical analysis

Prism 9 software (GraphPad) was used to perform statistical analysis. One-way ANOVA with Tukey’s multiple-comparisons test was used to determine the significance of differences among group means. Fisher’s exact test was used to determine the significance of differences in incidence of hydrosalpinx or granulomatous inflammation between genotypes. The Mann-Whitney U test was used for determining statistical significance for nonparametric data. All graphs represent mean plus or minus standard error of the mean of at least three independent experiments.

## Data Availability

The numerical values of all quantified data depicted in data panels in this manuscript are openly available in the Digital Repositories at Duke at https://doi.org/10.7924/r4r214s79

## SUPPLEMENTAL MATERIAL

Figure S1 mbio.00303-24-s0001.docx Increased inflammation, tissue distortion, and pathology in Irgm- and Rag1-deficient *C. muridarum*-infected mice.

ASM does not own the copyrights to Supplemental Material that may be linked to, or accessed through, an article. The authors have granted ASM a non-exclusive, world-wide license to publish the Supplemental Material files. Please contact the corresponding author directly for reuse.
