# Supplementary material for: Irgm proteins attenuate inflammatory disease in mouse models of genital Chlamydia infection
Source: mBio. 2024 Mar 19;15(4):e00303-24. doi: 10.1128/mbio.00303-24 (PMC11005385; doi:10.1128/mbio.00303-24)
Supplement: Figure S1 — Increased inflammation, tissue distortion, and pathology in Irgm- and Rag1-deficient C. muridarum-infected mice. [file mbio.00303-24-s0001.docx]

## Supplementary Data

**Figure S1**


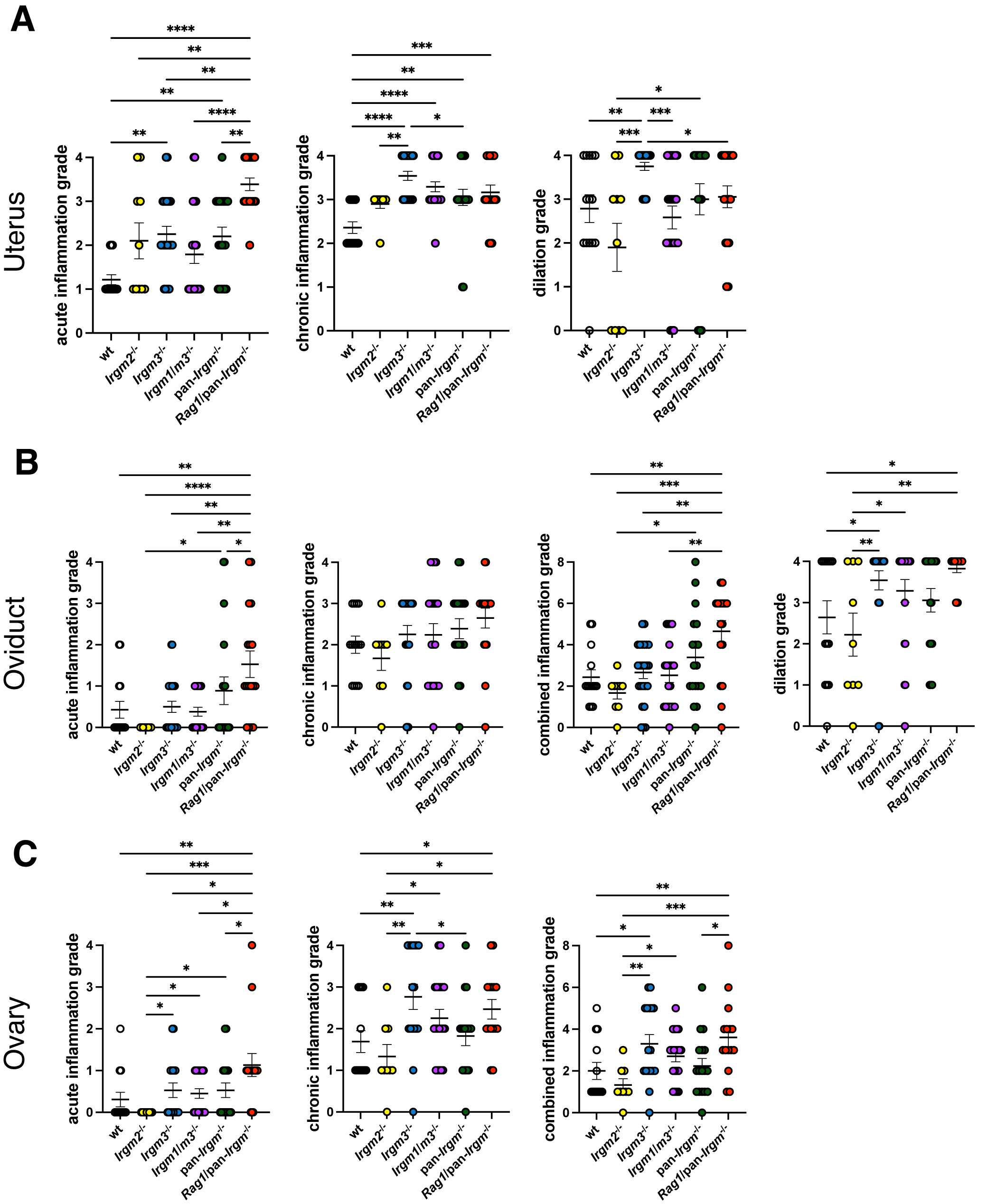


**Figure S1. Increased inflammation, tissue distortion, and pathology in Irgm- and Rag1-deficient *C. muridarum* infected mice**

Mice were infected transcervically with 2.5x10^5^ *C. muridarum* EBs. Genital tracts were harvested at 45 dpi, fixed, sectioned, H&E stained, and graded by a veterinary pathologist. Graphs represent the magnitude of acute inflammation, chronic inflammation, combined acute and chronic inflammation, and tissue dilation as defined by standard scoring criteria in the uteri **(A)**, oviducts **(B)**, and ovaries **(C)** of infected mice. Graphs represent pooled data from two independent experiments (wildtype n = 7 mice, *Irgm2*^-/-^ n = 5, *Irgm3*^-/-^ n = 12, *Irgm1*/*m3*^-/-^ n = 12, pan-*Irgm*^-/-^ n = 10, *Rag1*/pan*-Irgm*^-/-^ n = 9; each data point represents one uterine horn of an infected mouse). Statistical significance was determined using Mann-Whitney tests; * p<0.05, ** p<0.005, *** p<0.0005, **** p<0.00005; all comparisons not indicated as significant were not statistically significant
